# Aldol Reactions of Axially Chiral 5-Methyl-2-(*o*-aryl)imino-3-(*o*-aryl)-thiazolidine-4-ones

**DOI:** 10.3390/molecules21060788

**Published:** 2016-06-18

**Authors:** Sule Erol Gunal, Ilknur Dogan

**Affiliations:** Chemistry Department, Bogazici University, Bebek, 34342 İstanbul, Turkey; sule.erol@boun.edu.tr

**Keywords:** chromatographic separations on a chiral stationary phase, axial chirality, aldol reactions of thiazolidine-4-ones

## Abstract

Axially chiral 5-methyl-2-(*o*-aryl)imino-3-(*o*-aryl)-thiazolidine-4-ones have been subjected to aldol reactions with benzaldehyde to produce secondary carbinols which have been found to be separable by HPLC on a chiral stationary phase. Based on the reaction done on a single enantiomer resolved via a chromatographic separation from a racemic mixture of 5-methyl-2-(α-naphthyl)imino-3-(α-naphthyl)-thiazolidine-4-one by HPLC on a chiral stationary phase, the aldol reaction was shown to proceed via an enolate intermediate. The axially chiral enolate of the thiazolidine-4-one was found to shield one face of the heterocyclic ring rendering face selectivity with respect to the enolate. The selectivities observed at C-5 of the ring varied from none to 11.5:1 depending on the size of the *ortho* substituent. Although the aldol reaction proceeded with a lack of face selectivity with respect to benzaldehyde, recrystallization returned highly diastereomerically enriched products.

## 1. Introduction

Synthesis of axially chiral biaryls has received particular attention lately due to the presence of these scaffolds in a large number of natural products including some antibiotics [1]. Reactions of nonbiaryl axially chiral compounds on the other hand are relatively unexploited, although they have been shown to transmit stereochemical information for asymmetric induction [2]. Curran has shown that [3] several thermal reactions of axially chiral amides and imides are stereoselective because one face of the axially chiral molecule was shielded by the bulky *ortho* substituent. Recently, an atropisomerism induced facial selectivity was observed in cycloaddition reactions with 5-methylene hydantoins [4]. Other reactions have been carried out where the formation of new centers of chirality was controlled by an axis of chirality [5,6,7,8,9,10,11,12].

**Scheme 1:**
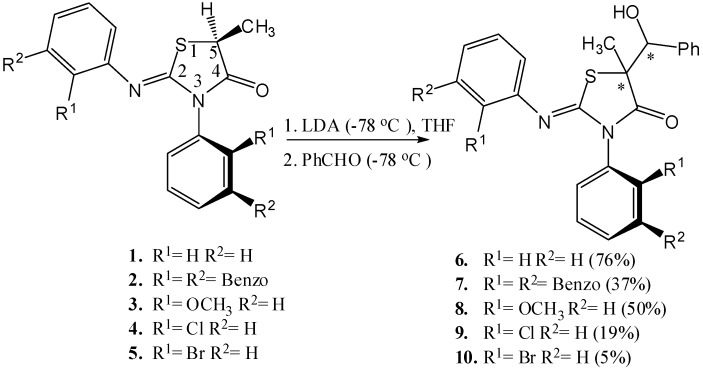
The aldol reactions of compounds **1**–**5** (% yields after purification by recrystallization are reported in *parentheses*).

We recently disclosed the diastereoselective synthesis of 2-(*o*-aryl)imino-3-(*o*-aryl)-5-methyl-thiazolidine-4-ones [13] (Scheme 1) bearing an axis of chirality and showed that they are conformationally and configurationally stable even at room temperature [13,14]. In this paper, we report the aldol reactions of **1**–**5** (Scheme 1) which were found to proceed via enolates possessing an axis of chirality, to produce chiral secondary carbinols separable by HPLC on a chiral sorbent. The face selectivity of the axially chiral enolate intermediate enabled a diastereoselective synthesis.

## 2. Results and Discussion

**Scheme 2:**
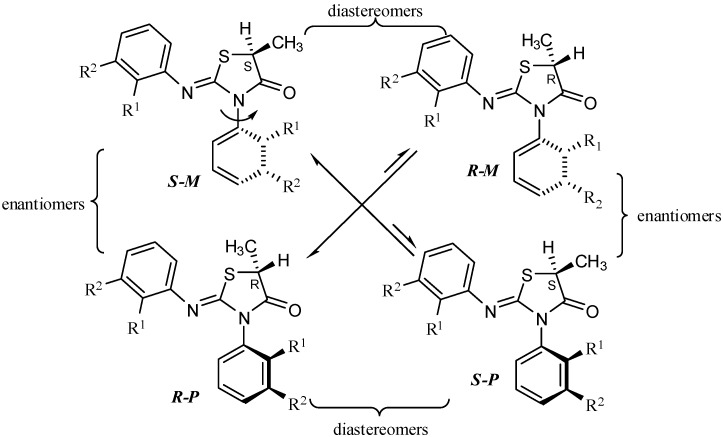
The stereoisomers of 5-methyl-2-arylimino-3-*o*-aryl-thiazolidine-4-ones.

Compounds **2**–**5** contain both a stereogenic center at C-5 of the heterocyclic ring and an axis of chirality [14] due to the hindered rotation around the N_sp2_-C_aryl_ bond and therefore exist as two diastereomeric pairs [13,15] (Scheme 2). The aldol reactions [16] of compounds **1**–**5** were carried out by treating the compounds with lithium diisopropyl amide (LDA) at −78 °C and followed by an electrophile quench with benzaldehyde (at −78 °C) (Scheme 1).

**Figure 1:**
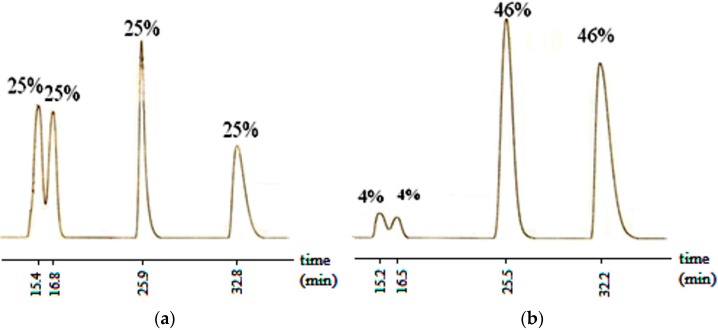
HPLC chromatogram of **6** showing the isomer compositions (**a**) at the end of the reaction; and (**b**) after recrystallization from ethyl acetate-hexane. (Column: Chiralpak IB, Eluent (*v*/*v*) hexane: ethanol (95:5), flow rate: 0.7 mL/min).

Aldol reaction of the compound **1** which lacks an axis of chirality was initially investigated. The aldol product of it (**6**) contains two centers of chirality and the number of possible isomers is four (*RR*, *SS*, *RS* and *SR*). Figure 1a shows the % compositions of the obtained isomers analyzed immediately after the reaction, on Chiralpak IB column by HPLC, before purification. The HPLC trace indicates that the reaction proceeds with a lack of face selectivity of benzaldehyde producing equal ratios of all isomers. However, recrystallization from ethyl acetate-hexane returned a diastereomeric ratio of 92:8 (Figure 1b). Although this reaction was not diastereoselective, the diastereomeric pairs turned out to be separable by recrystallization.

**Figure 2:**
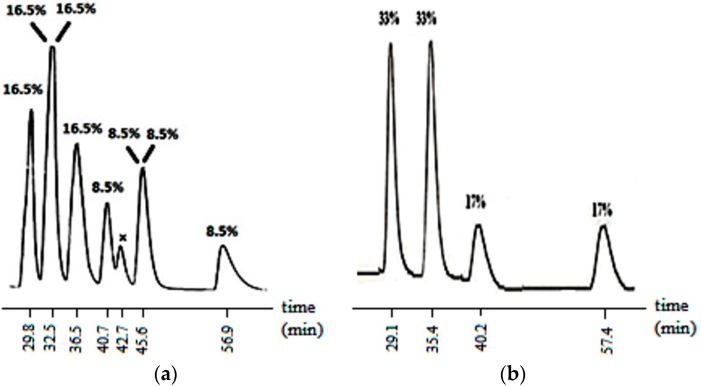
HPLC chromatogram of **7** showing the isomer compositions (**a**) at the end of reaction (without purification, x: impurity ^a^; (**b**) when the aldol reaction was carried out on a single stereoisomer of **2**. (Column: Chiralpak IB, Eluent (*v*/*v*) hexane:ethanol: 95:5, flow rate: 0.7 mL/min). ^a^ X was confirmed to be an impurity because it was absent in the HPLC of the purified product.

The aldol reaction was then carried out on compound **2** which had a diastereomeric composition of 72:28. With the introduction of an axis of chirality, the expected number of possible stereoisomers of the aldol product increased to eight. In fact, at the end of the reaction, when the crude reaction mixture was analyzed by HPLC without purification, the six of the eight stereoisomers were found to be separable on a Chiralcel IB column and two of them coincided (Figure 2a).

**Scheme 3:**
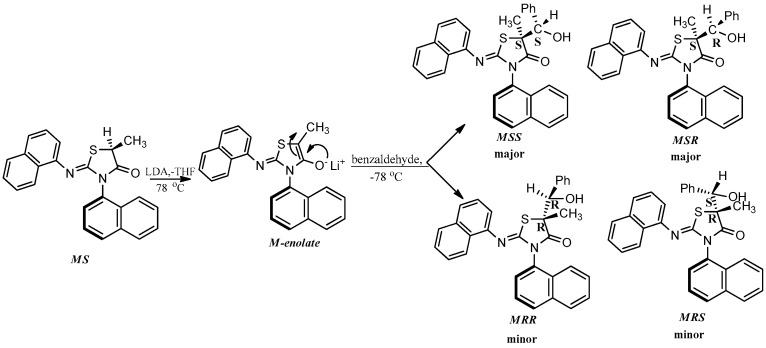
The aldol reaction carried out on a single isomer of the compound **2** via an enolization mechanism.

**Figure 3:**
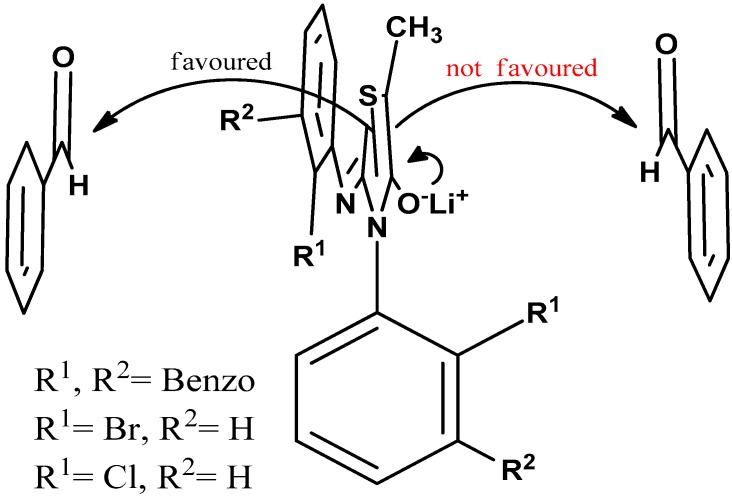
The attack of the axially chiral racemic enolate to the electrophile is favored by the unhindered face rendering face selectivity.

The enolate of **2** (Scheme 3) has two diastereotopic faces (Figure 3), one of which can be expected to be shielded by the *ortho* substituent of the aryl group on N_3_ (Figure 3) rendering face selectivity. The aldol reaction was also carried out on a single enantiomer of **2** obtained by a micropreparative resolution of the racemic **2** by HPLC on Chiralpak IB. The aldol reaction done on a single enantiomer was found to yield the product that consisted of four stereoisomers (Figure 2b).

The isomer distribution of the aldol products of the single enantiomer of **2** showed the presence of two major (*MSS* and *MSR*) and two minor (*MRR* and *MRS*) isomers (Figure 2b, Scheme 3). This pointed to a face selective reaction where the chirality axis of the enolate controlled the configuration of the carbon at 5-position of the heterocyclic ring. However, no facial selectivity was observed with respect to the addition of benzaldehyde. Thus, *MSS* and *MSR* (or *MRR* and *MRS*) were formed in equal amounts. The ratio of major to minor isomers was found to be ~2:1 (Figure 2b) indicating a diastereoselectivity of 2:1.

The aldol reactions were also carried out on compounds **3**, **4** and **5** having *ortho* OCH_3_, Cl and Br substituents, respectively. The steric requirement for the OCH_3_ group is much lower than those of the Cl and Br, as evident from the barriers to hindered rotations caused by them as *ortho* substituents [13,14]. Therefore, although no selectivity was observed for the *o*-methoxy derivative, as the size of the *ortho* substituent increased, it was able to shield one face of the enolate. For the compound with an *o*-Cl substituent, the corresponding selectivity was found to be 4:1 and it reached to 11.5:1 for compound with an *o*-Br substituent. The observed selectivities are listed in Table 1.

## 3. Experimental Section

### 3.1. General Information

^1^H- and ^13^C-nuclear magnetic resonance (NMR) spectra of all compounds were recorded on a Varian-Mercury VX-400 MHz- BB (Varian Co., Palo Alto, CA, USA). IR spectra of all compounds were recorded on Thermo Nicolet 380 FT-IR spectrometer (Thermo Fisher Scientific, Waltham, MA, USA). Liquid chromatography analyses with ultraviolet (UV) detector (λ = 254 nm) were performed using CHIRALPAK IB column (particle size, 5 mm; column size, 250 × 4.6 mm^2^). Elemental analyses were performed on Thermo Scientific Flash EA 1112 analyzer (Thermo Fisher Scientific, Waltham, MA, USA). Melting points were recorded using Electrothermal 9100 melting point apparatus (Electrothermal Bibby Scientific Ltd., Staffordshire, UK). All reagents and solvents were purchased from commercially available suppliers (Merck Darmstadt, Germany, Aldrich, St. Louis, MO, USA).

### 3.2. General Procedure for Aldol Reactions

Reactions were carried out under nitrogen. To a solution of 5-methyl-2-(*o*-aryl)imino-3-(*o*-aryl)-thiazolidine-4-ones (**1**–**5**) in dry THF (obtained from JC Meyer solvent drying system) at −78 °C was added lithium diisopropylamide (LDA) (2 M, 1.2 equiv.). The reaction mixture was stirred for 1 h for enolate formation at −78 °C. Then, benzaldehyde (2.0 equiv.) was added. The reaction mixture was stirred for 4 h at −78 °C and then quenched by adding saturated ammonium chloride solution (2 mL/mmol of thiazolidine-4-one used). The mixture was extracted immediately three times with 10 mL diethyl ether. One milliliter of this solution was taken, dried over MgSO_4_ and quickly injected to the chiral column after evaporation of the ether to determine the crude product distribution. The remaining organic layer was then dried, the volatiles evaporated and the crude product was recrystallized from ethyl acetate-hexane. See Figures S3–S12 in the Supplementary Materials for the NMR spectra.

*5-(1-Hydroxybenzyl)-5-methyl-2-(phenyl)imino-3-(phenyl)-thiazolidine-4-one* (**6**): The compound was synthesized according to the general procedure using compound **1** (0.34 g, 1.2 mmol) in dry THF (10 mL), LDA (0.36 mL, 1.44 mmol) and benzaldehyde (0.12 mL, 2.4 mmol). Yield: 0.2 g (76%), mp: 176–178 °C. ^1^H-NMR (400 MHz, CDCl_3_ for one diastereomer): δ 6.93–7.53 (15H, m, ArH), 5.15 (1H, s, C*H*OH), 2.67 (1H, d, *J =* 4.0 Hz, OH), 1.61 (1H, s, CH_3_) ppm. ^13^C-NMR (100 MHz, CDCl_3_): δ 176.7, 154.5, 148.4, 138.5, 135.0, 129.3, 129.1, 129.0, 128.9, 128.8, 128.4, 128.0, 127.9, 124.4, 121.1, 120.8, 62.4, 24.5 ppm. Calculated for C_23_H_20_N_2_O_2_S: C, 71.11; H, 5.19; N, 7.21; S, 8.25. Found: C, 71.25; H, 5.05; N, 7.16; S, 8.60. HRMS (TOF MS ES+) [M + Na]^+^ calculated for C_23_H_20_N_2_O_2_S 411.1143; found 411.1157. FTIR (neat) 3374 (OH), 1633 (C=O), 1592 (C=N) cm^−1^.

*5-(1-Hydroxybenzyl)-5-methyl-2-(α-naphthyl)imino-3-(α-naphthyl)-thiazolidine-4-one* (**7**): The compound was synthesized according to the general procedure using compound **2** (0.46 g, 1.2 mmol) in dry THF (10 mL), LDA (0.36 mL, 1.44 mmol) and benzaldehyde (0.12 mL, 2.4 mmol). Yield: 0.21 g (37%), mp: 170–172 °C.^1^H-NMR (400 MHz, CDCl_3_ for one diastereomer): δ 7.07–8.20 (19H, m, ArH), 5.25 (1H, s, C*H*OH), 2.76 (1H, d, *J* = 3.7 Hz, OH), 1.65 (3H, s, CH_3_) ppm. ^13^C-NMR (100 MHz, CDCl_3_): δ 177.0, 153.8, 144.7, 138.5, 134.7, 134.2, 132.4, 130.2, 130.1, 128.9, 128.5, 128.4, 128.3, 127.7, 127.6, 127.5, 127.3, 126.8, 126.6, 126.1, 125.6, 125.5, 125.4, 124.6, 123.7, 123.1, 115.1, 112.6, 112.3, 112.1, 63.3, 24.6 ppm. Calculated for C_31_H_24_N_2_O_2_S: C, 76.20; H, 4.95; N, 5.73; S, 6.56 ppm. Found: C, 75.93; H, 4.98; N, 5.60; S, 6.32. HRMS (TOF MS ES+) [M + Na]^+^ calculated for C_31_H_24_N_2_O_2_S 511.1456; found 511.1460.

*5-(1-Hydroxybenzyl)-5-methyl-2-(o-methoxyphenyl)imino-3-(o-methoxyphenyl)-thiazolidine-4-one* (**8**): The compound was synthesized according to the general procedure using compound **3** (0.41g, 1.2 mmol) in dry THF (10 mL), LDA (0.36 mL, 1.44 mmol) and benzaldehyde (0.12 mL, 2.4 mmol). Yield: 0.26 g (50%), mp: 168–170 °C. ^1^H-NMR (400 MHz, CDCl_3_): δ 6.64–7.43 (13H, m, ArH), 5.10 (1H, s, C*H*OH), 3.79 (3H, s, OCH_3_), 3.69 (3H, s, OCH_3_), 1.44 (3H, s, CH_3_) ppm. ^13^C-NMR (100 MHz, CDCl_3_): δ 177.6, 176.4, 154.9, 153.4, 138.1, 137.9, 137.5, 130.9, 130.7, 129.9, 129.7, 129.6, 128.7, 128.5, 128.2, 128.0, 127.9, 127.8, 125.3, 125.2, 123.3, 121.8, 121.7, 121.0, 120.8, 61.8, 60.1, 56.0, 59.93, 59.88, 55.84, 23.1, 21.2 ppm. Calculated for C_25_H_24_N_2_O_4_S: C, 66.94; H, 5.39; N, 6.25; S, 7.15. Found: C, 66.34; H, 5.30; N, 6.44; S, 7.53. HRMS (TOF MS ES+) [M + Na]^+^ calculated for C_25_H_24_N_2_O_4_S; found 471.1354; found 471.1384. 

*5-(1-Hydroxybenzyl)-5-methyl-2-(o-chlorophenyl)imino-3-(o-chlorophenyl)-thiazolidine-4-one* (**9**): The compound was synthesized according to the general procedure using compound **5** (0.42 g, 1.2 mmol) in dry THF (10 mL), LDA (0.36 mL, 1.44 mmol) and benzaldehyde (0.12 mL, 2.4 mmol). Yield: 0.1 g (19%), mp: 54–56 °C. ^1^H-NMR (400 MHz, CDCl_3_): δ 7.51–6.49 (13H, m, ArH), 5.15 (1H, s, C*H*OH), 5.12 (1H, s, C*H*OH), 5.10 (1H, s, C*H*OH), 1.94 (3H, s, CH_3_), 1.84 (3H, s, CH_3_), 1.57 (3H, s, CH_3_) ppm. ^13^C-NMR (100 MHz, CDCl_3_): δ 176.0, 156.0, 144.5, 138.3, 137.9, 132.8, 132.6, 130.8, 130.7, 130.6, 130.5, 130.4, 130.2, 130.1, 130.0, 129.9, 128.9, 128.8, 128.4, 128.1, 127.9, 127.3, 126.1, 125.5, 125.4, 122.1, 63.8, 29.8, 25.4, 25.0, 24.0 ppm. Calculated for C_23_H_18_Cl_2_N_2_O_2_S: C, 60.40; H, 3.97; N, 6.12; S, 7.01. Found: C, 60.37; H, 3.51; N, 6.19; S, 7.38. HRMS (TOF MS ES+) [M + Na]^+^ calculated for C_23_H_18_Cl_2_N_2_O_2_S 479.0364; found 479.0362.

*5-(1-Hydroxybenzyl)-5-methyl-2-(o-bromophenyl)imino-3-(o-bromophenyl)-thiazolidine-4-one* (**10**): The compound was synthesized according to the general procedure using compound **5** (0.046 g, 0.16 mmol) in dry THF (2 mL), LDA (0.09 mL, 0.19 mmol) and benzaldehyde (0.03 mL, 0.32 mmol). Yield: 0.04 g (5%), mp: 68–70 °C. ^1^H-NMR (400 MHz, CDCl_3_ for four diastereomeric pairs): δ 7.69–6.50 (13H, m, ArH), 5.21 (1H, s, C*H*OH), 5.20 (1H, s, C*H*OH), 5.13 (1H, s, C*H*OH), 5.10 (1H, s, C*H*OH), 2.10 (3H, s, CH_3_), 1.96 (3H, s, CH_3_), 1.81 (3H, s, CH_3_), 1.58 (3H, s, CH_3_) ppm. ^13^C-NMR (100 MHz, CDCl_3_): δ 172.0, 138.3, 133.9, 133.8, 133.4, 131.1, 130.8, 130.4, 128.9, 128.9, 128.7, 128.5, 128.4, 127.7, 125.9, 123.1, 122.0, 121.8, 116.2, 64.0, 64.1, 24.0, 24.5 ppm. Calculated for C_23_H_18_Br_2_N_2_O_2_S: C, 50.57; H, 3.32; N, 5.13; S, 5.87. Found: C, 51.30; H, 3.51; N, 5.65; S, 5.45.

## 4. Conclusions

The aldol reactions of 5-methyl-2-(*o*-aryl)imino-3-(*o*-aryl)-thiazolidine-4-ones which possess an axis of chirality have been found to proceed via the axially chiral ***M*** and ***P*** enolates where one face of the enolate could be shielded by the substituent at the *ortho* position of the N_3_-aryl ring. The resulting selectivities were found to be up to 11.5:1. The aldol reactions were found to proceed with a lack of face selectivity of benzaldehyde, but recrystallization returned highly diastereomerically enriched products.

## Figures and Tables

**Table 1:** The diastereoselectivities ^a^ observed before and after recrystallization.

Compounds	Before Recrystallization ^b^	After Recrystallization ^c^
**7**	66:34	7:93
**8**	50:50	97:3
**9**	80:20	76:24
**10**	92:8	92:8

^a^ At C-5 position; ^b^ Determined by chiral HPLC at the end of the reaction before purification; ^c^ Recrystallized from ethyl acetate-hexane.

## Supplementary Materials

Supplementary materials can be accessed at: http://www.mdpi.com/1420-3049/21/6/788/s1.
