# Supplementary material for: Aldol Reactions of Axially Chiral 5-Methyl-2-(o-aryl)imino-3-(o-aryl)-thiazolidine-4-ones
Source: Molecules. 2016 Jun 18;21(6):788. doi: 10.3390/molecules21060788 (PMC6274475; doi:10.3390/molecules21060788)
Supplement: Supplementary file 1 [file molecules-21-00788-s001.pdf]

Supplementary Materials: Aldol Reactions of Axially Chiral 5-Methyl-2-(*o*-aryl)imino-3-(*o*-aryl)-thiazolidine-4-ones

Sule Erol Gunal and Ilknur Dogan

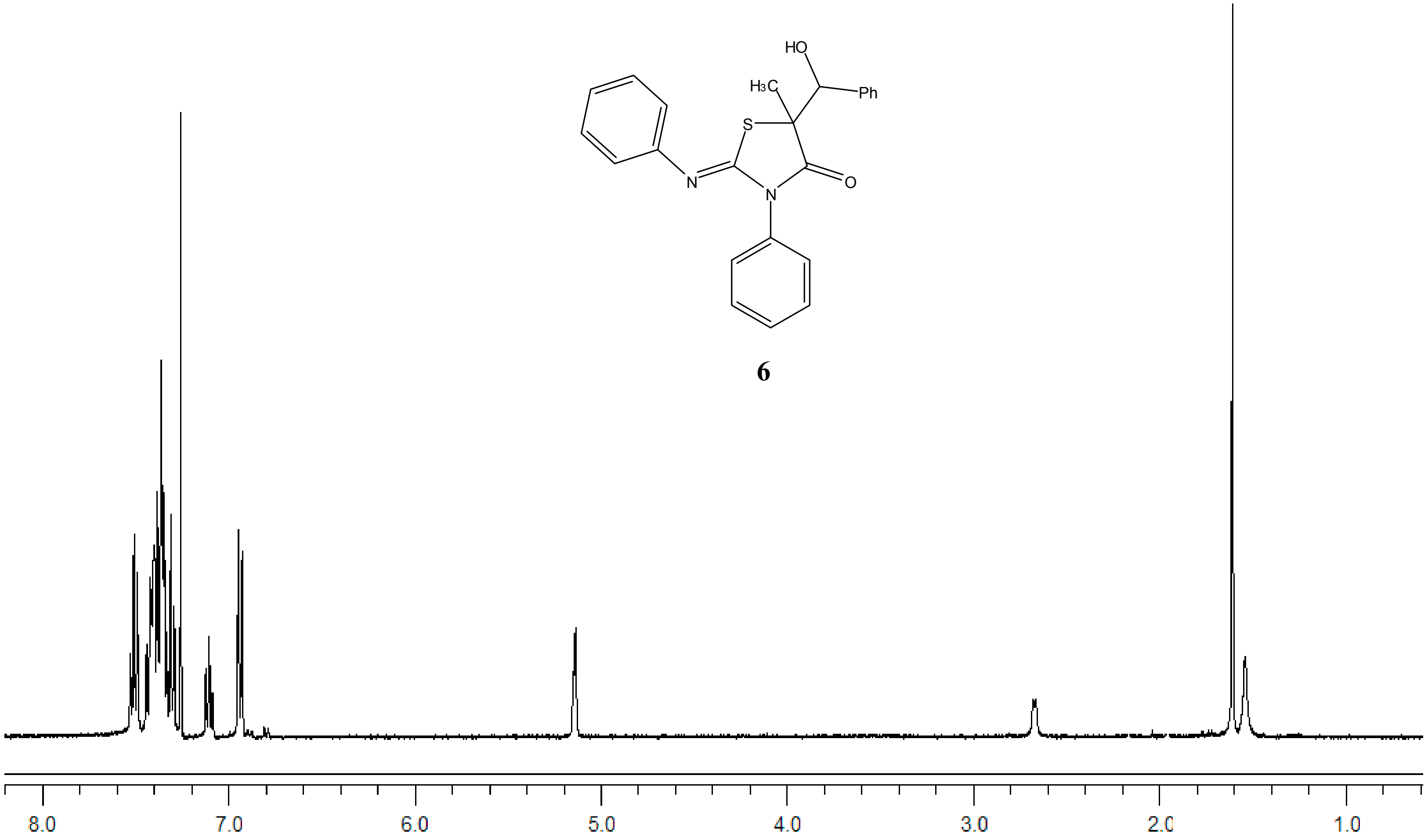

Figure S1. 400 MHz ^{1}H-NMR spectrum of 5-(1-Hydroxybenzyl)-5-methyl-2-(phenyl)imino-3-(phenyl)-thiazolidine-4-one (**6**) in $CDCl_3$.

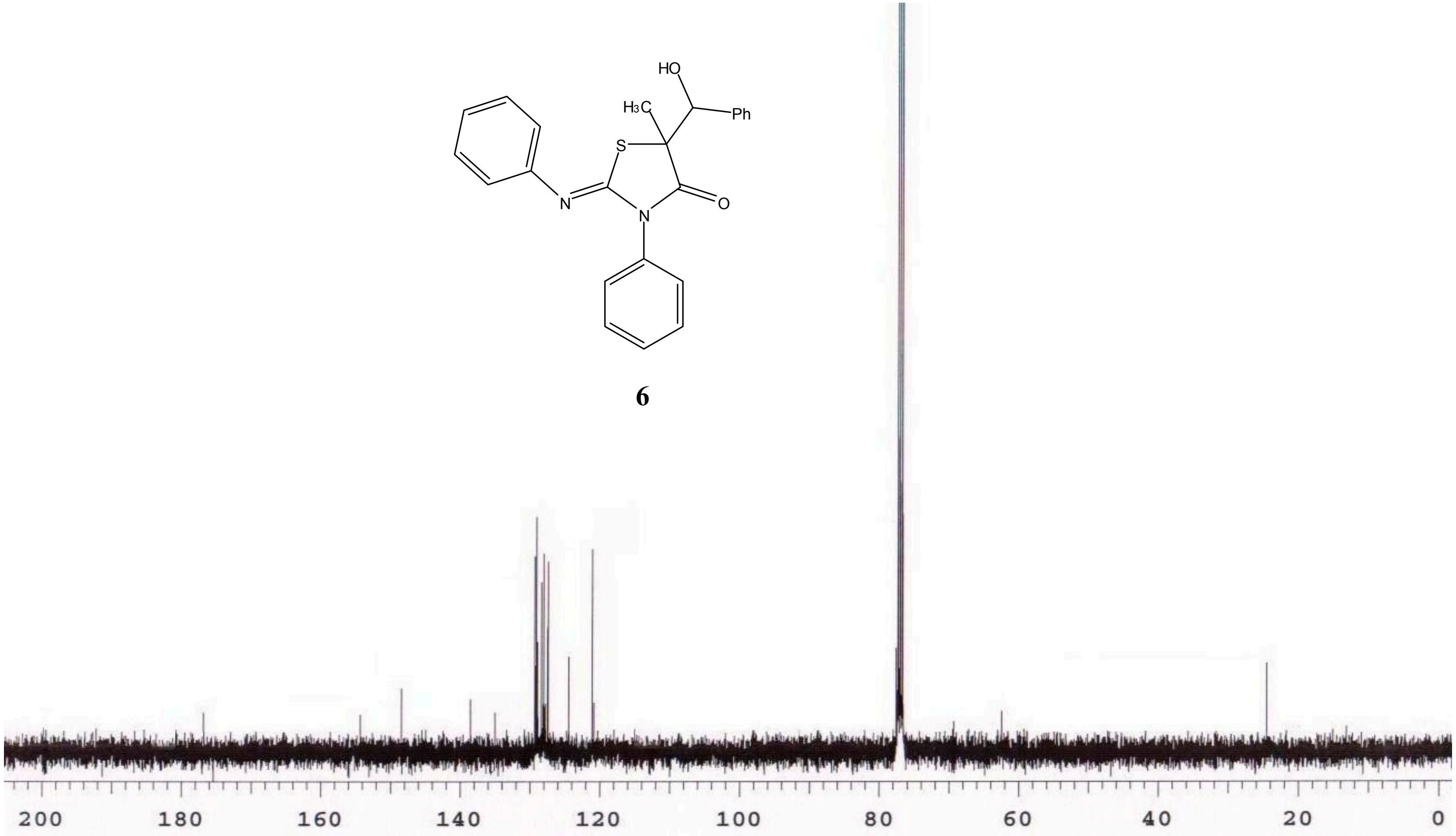

Figure S2. 100 MHz ^{13}C-NMR spectrum of 5-(1-Hydroxybenzyl)-5-methyl-2-(phenyl)imino-3-(phenyl)-thiazolidine-4-one (**6**) in $CDCl_3$.

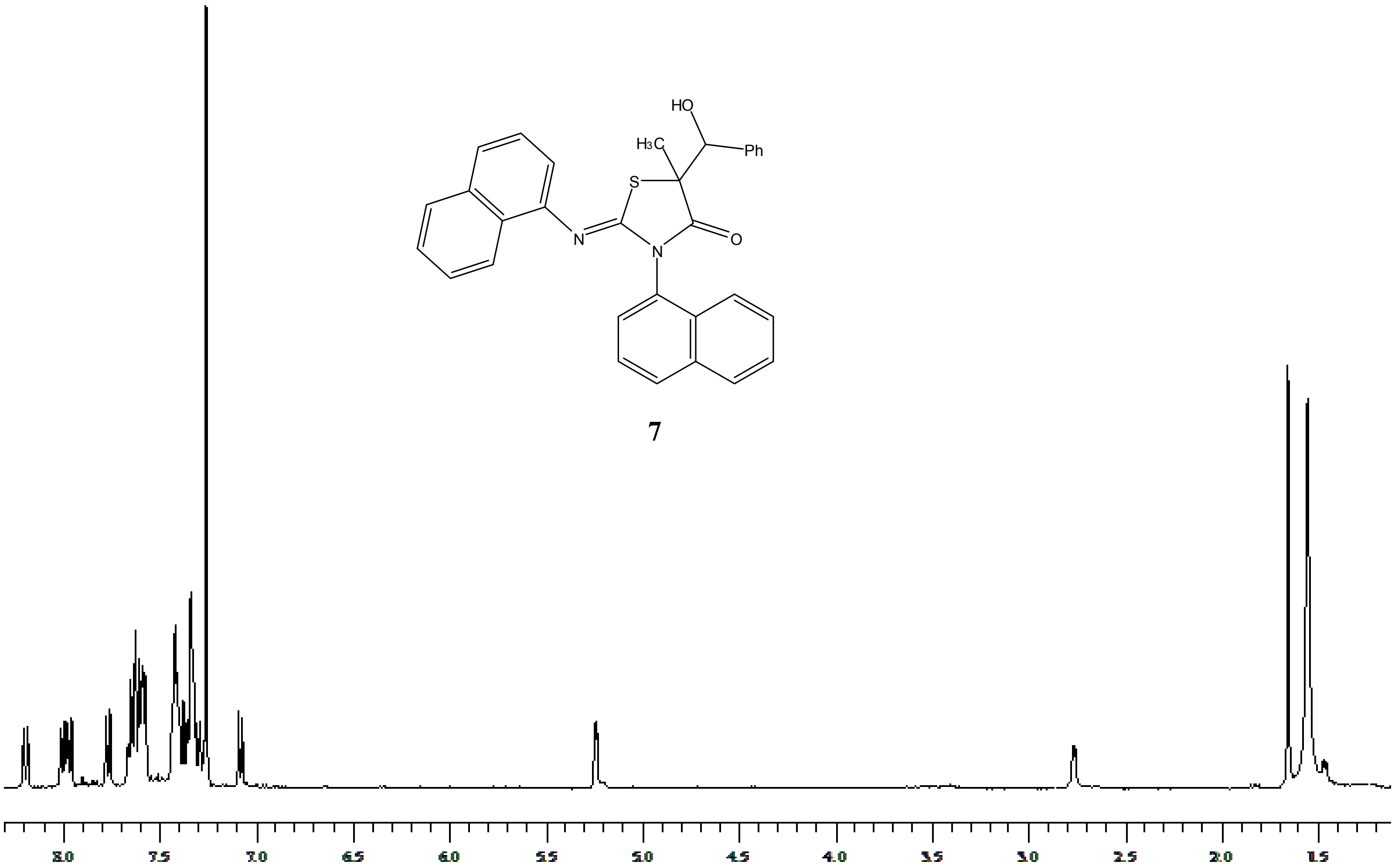

Figure S3. 400 MHz ^{1}H-NMR spectrum of 5-(1-Hydroxybenzyl)-5-methyl-2-(α-naphthyl)imino-3-(α-naphthyl)-thiazolidine-4-one (**7**) in $CDCl_3$.

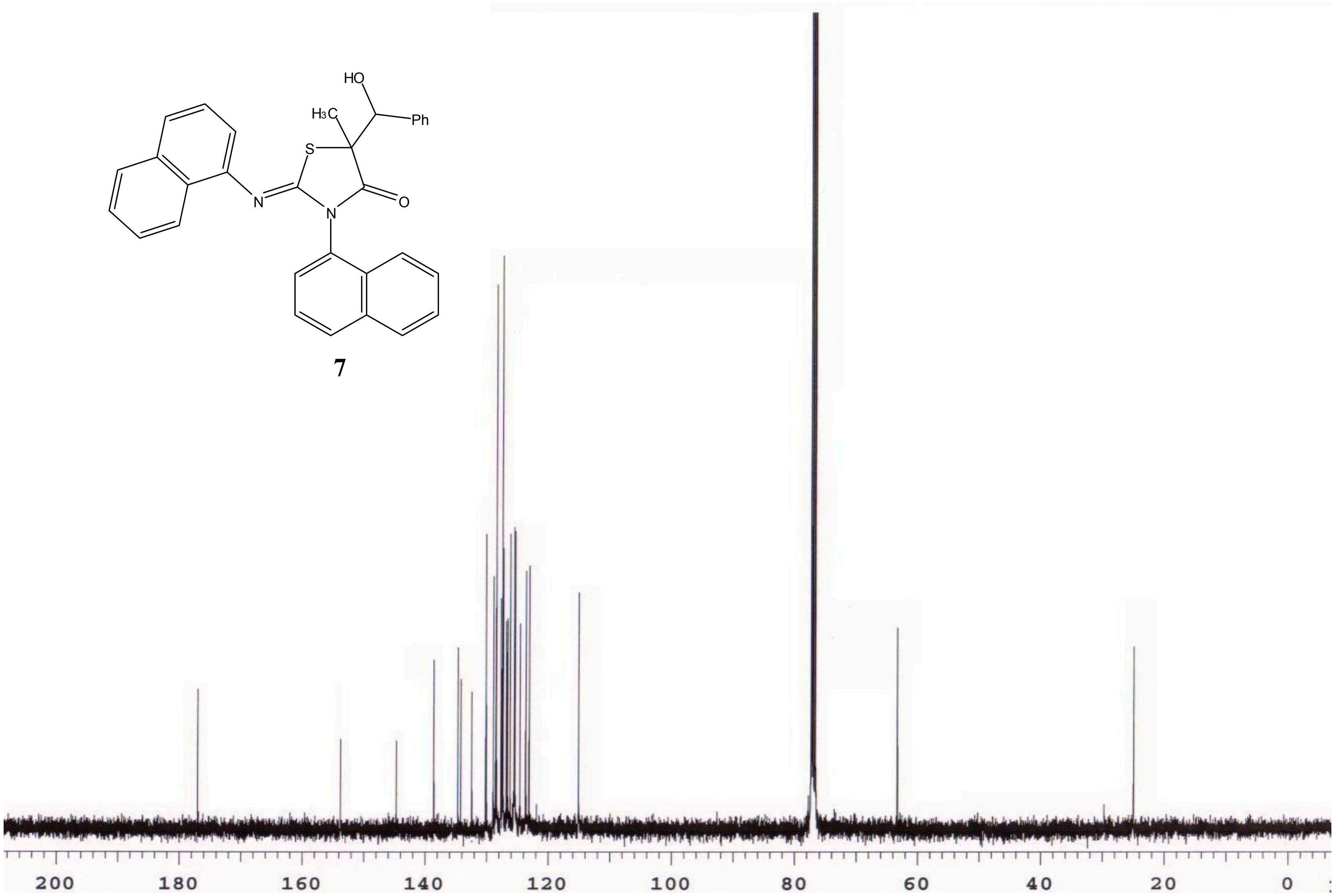

Figure S4. 100 MHz ^{13}C-NMR spectrum of 5-(1-Hydroxybenzyl)-5-methyl-2-(α-naphthyl)imino-3-(α-naphthyl)-thiazolidine-4-one (**7**) in $CDCl_3$.

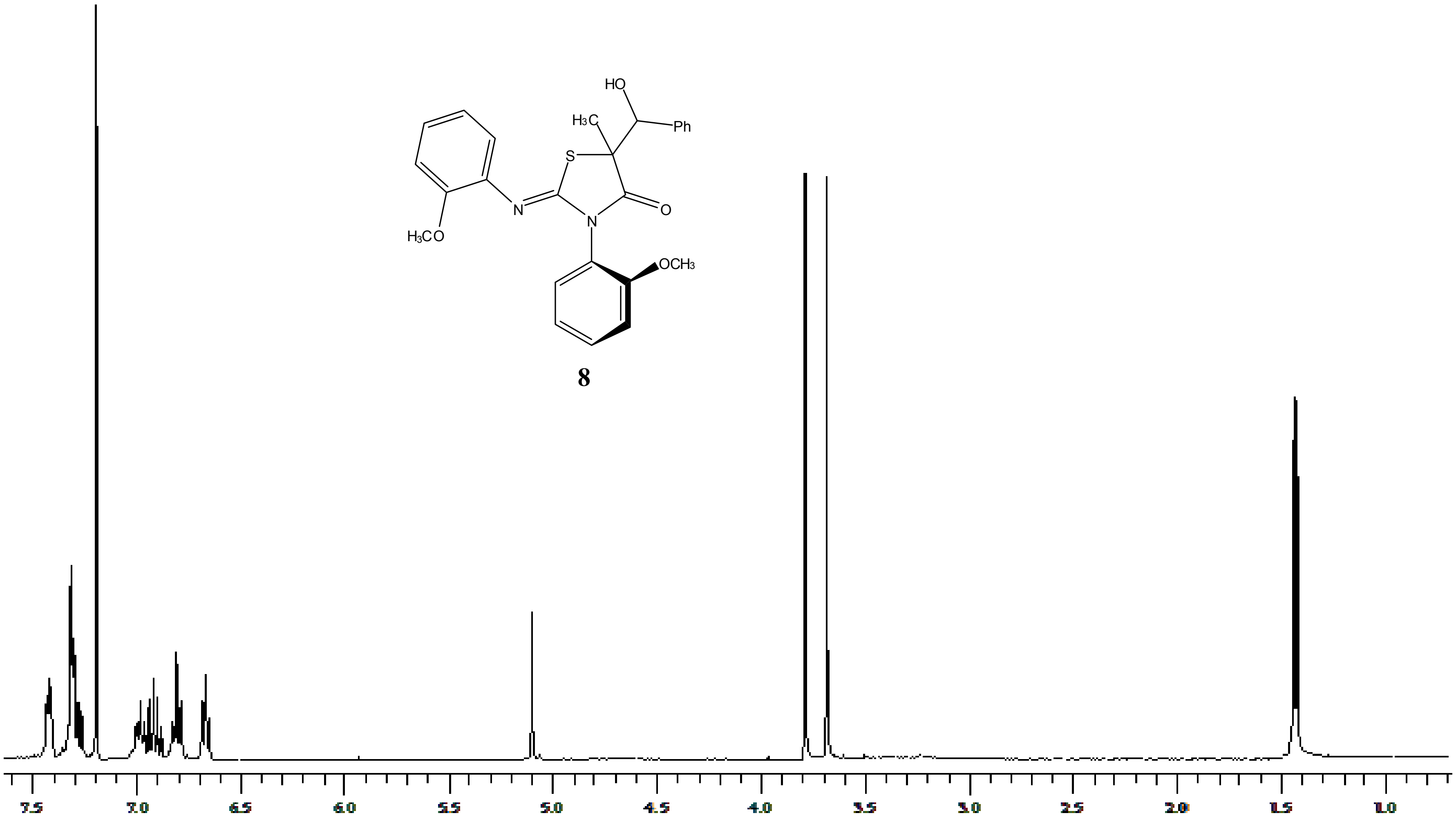

Figure S5. 400 MHz ^{1}H-NMR spectrum of 5-(1-Hydroxybenzyl)-5-methyl-2-(*o*-methoxyphenyl)imino-3-(*o*-methoxyphenyl)-thiazolidine-4-one (**8**) in $CDCl_3$.

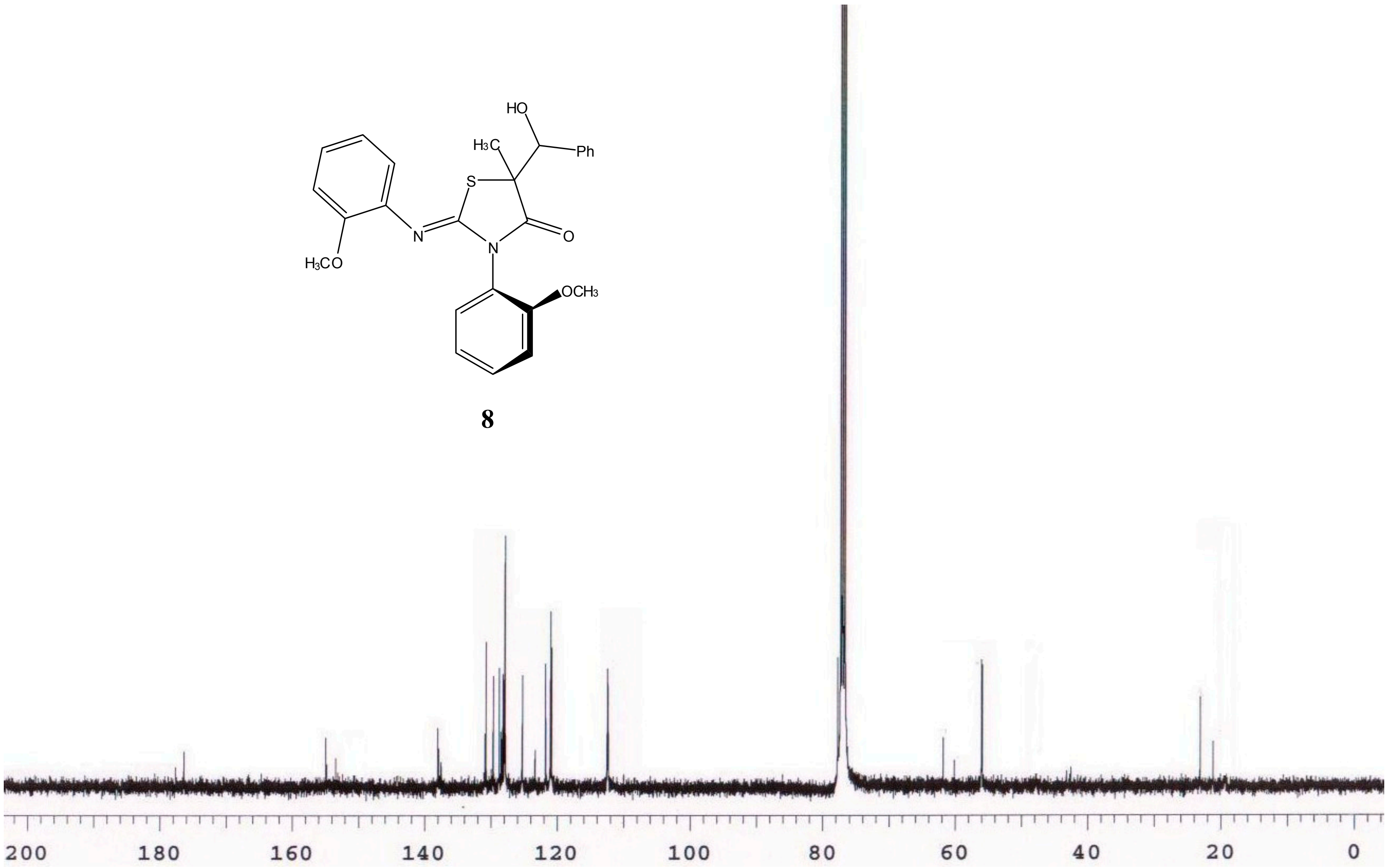

Figure S6. 100 MHz ^{13}C-NMR spectrum of 5-(1-Hydroxybenzyl)-5-methyl-2-(*o*-methoxyphenyl)imino-3-(*o*-methoxyphenyl)-thiazolidine-4-one (**8**) in $CDCl_3$.

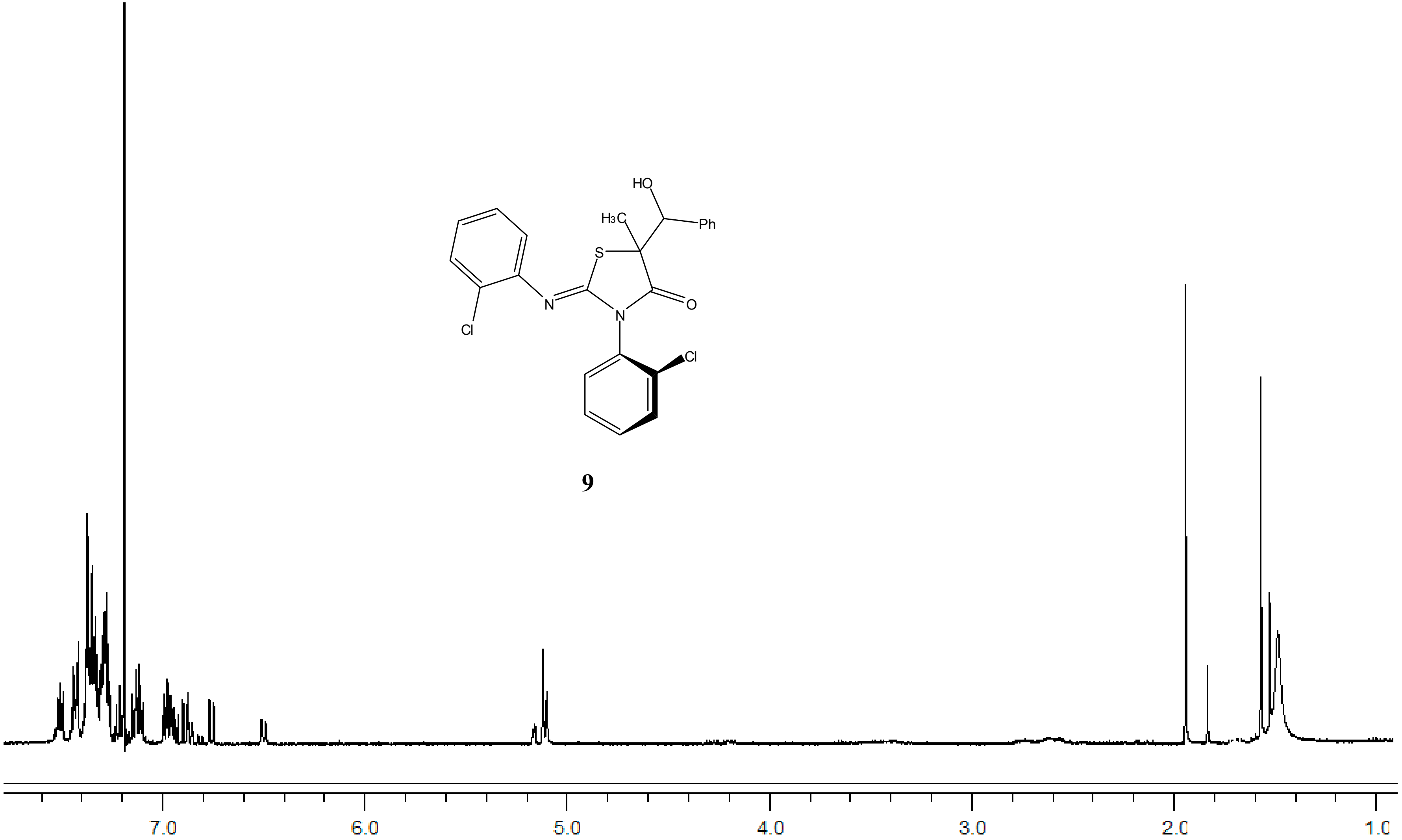

Figure S7. 400 MHz ^{1}H-NMR spectrum of 5-(1-Hydroxybenzyl)-5-methyl-2-(*o*-chlorophenyl)imino-3-(*o*-chlorophenyl)-thiazolidine-4-one (**9**) in $CDCl_3$.

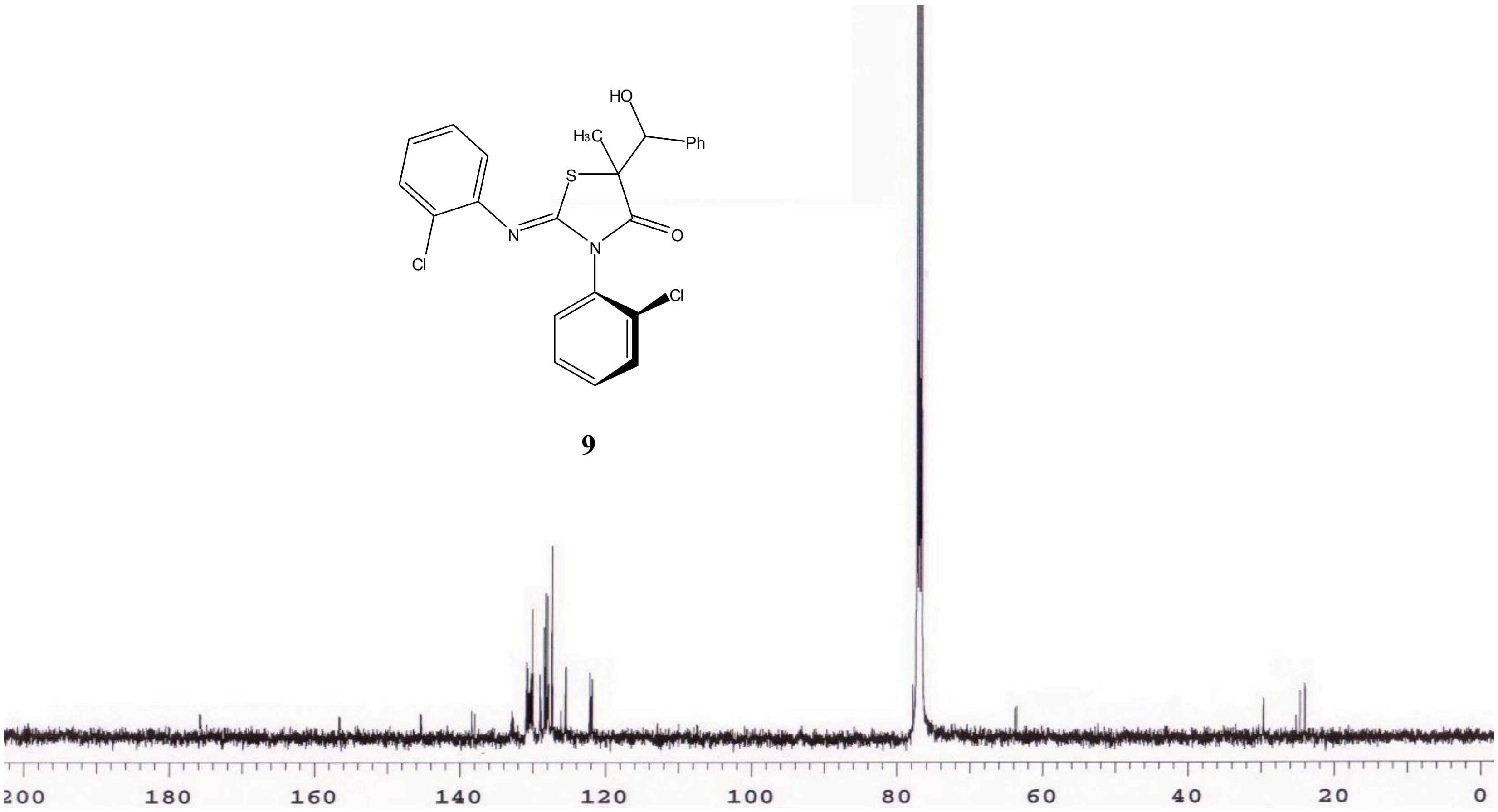

Figure S8. 100 MHz ^{13}C-NMR spectrum of 5-(1-Hydroxybenzyl)-5-methyl-2-(*o*-chlorophenyl)imino-3-(*o*-chlorophenyl)-thiazolidine-4-one (**9**) in $CDCl_3$.

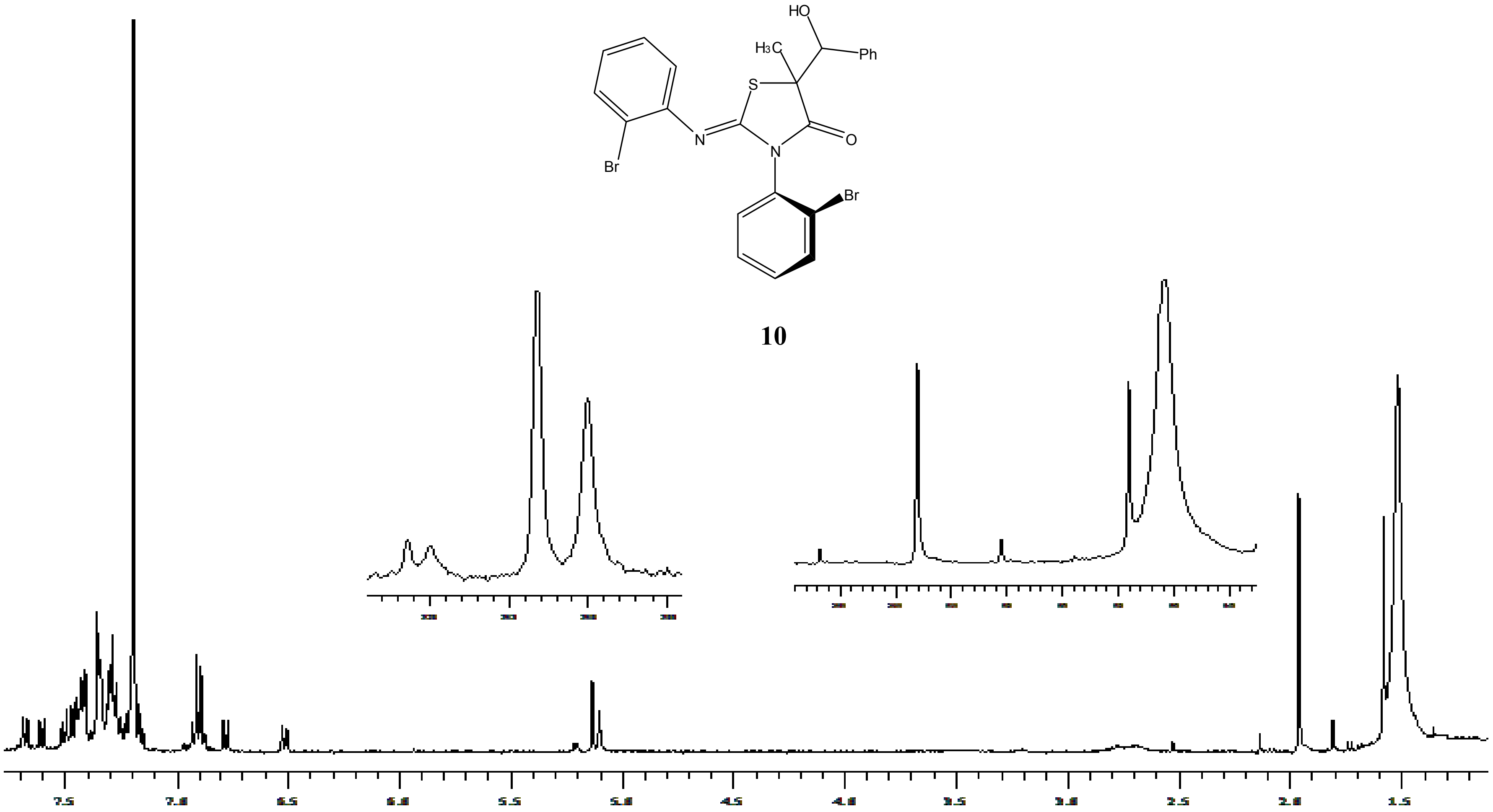

Figure S9. 400 MHz ^{1}H-NMR spectrum of 5-(1-Hydroxybenzyl)-5-methyl-2-(*o*-bromophenyl)imino-3-(*o*-bromophenyl)-thiazolidine-4-one (**10**) in $CDCl_3$.

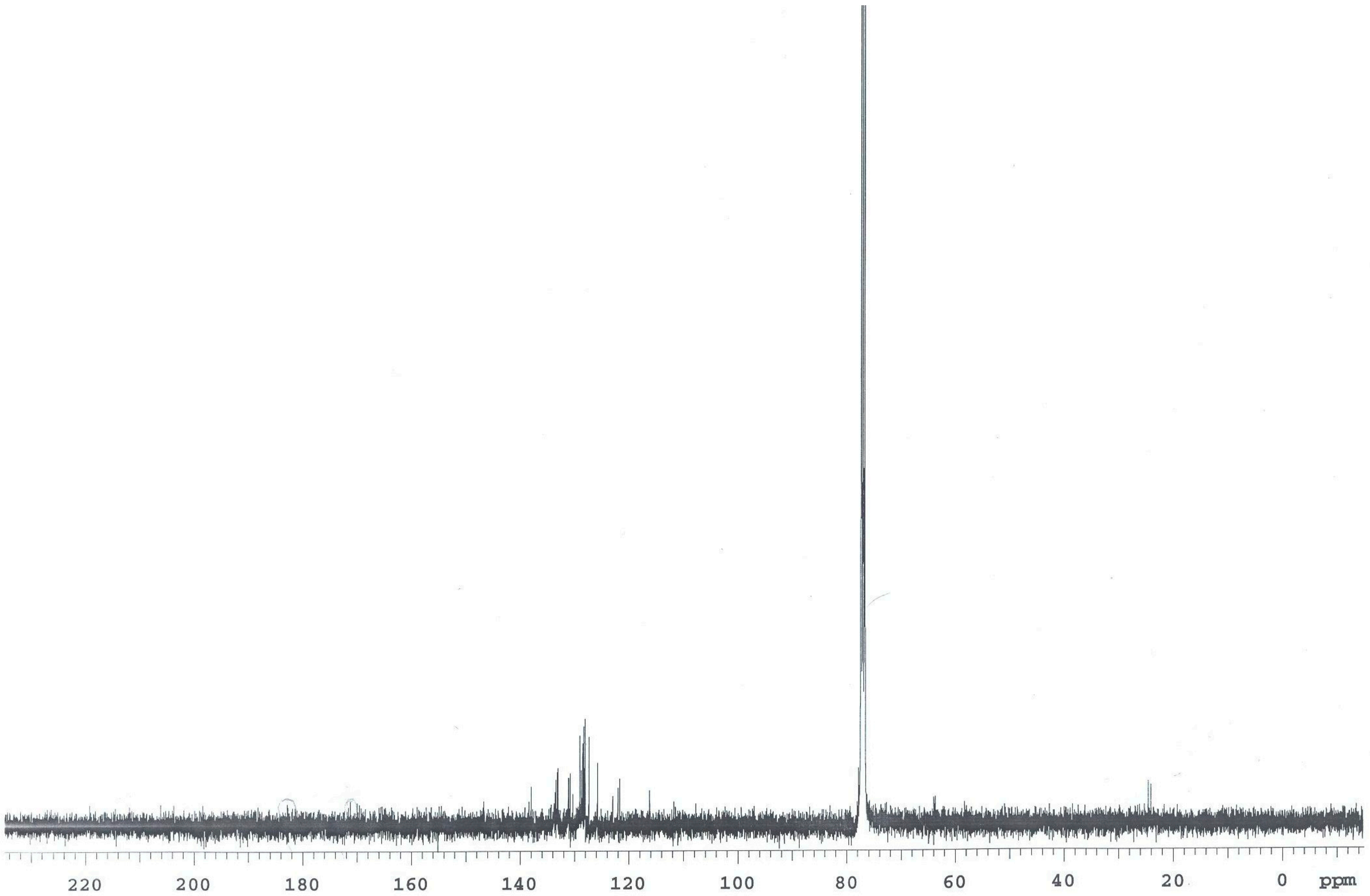

Figure S10. 100 MHz ^{13}C-NMR spectrum of 5-(1-Hydroxybenzyl)-5-methyl-2-(*o*-bromophenyl)imino-3-(*o*-bromophenyl)-thiazolidine-4-one (**10**) in $CDCl_3$.
